# Proteostasis in pediatric pulmonary pathology

**DOI:** 10.1186/s40348-014-0011-1

**Published:** 2014-12-29

**Authors:** Silke Meiners, Korbinian Ballweg

**Affiliations:** Comprehensive Pneumology Center (CPC), Member of the German Center for Lung Research (DZL), University Hospital, Ludwig-Maximilians-Universität, Asklepios Klinik Gauting und Helmholtz Zentrum München, Max-Lebsche-Platz 31, Munich, 81377 Germany

**Keywords:** Proteostasis, Conformational disorder, Autophagy, Proteasome, ER stress

## Abstract

Protein homeostasis describes the tight supervision of protein synthesis, correct protein maturation and folding, as well as the timely disposal of unwanted and damaged proteins by the ubiquitin-proteasome pathway or the lysosome-autophagy route. The cellular processes involved in preservation of protein homeostasis are collectively called proteostasis. Dysregulation of proteostasis is an emerging common pathomechanism for chronic lung diseases in the adult and aged patient. There is also rising evidence that impairment of protein homeostasis contributes to early sporadic disease onset in pediatric lung diseases beyond the well-known hereditary proteostasis disorders such as cystic fibrosis and alpha-1 antitrypsin (AAT) deficiency. Identifying the pathways that contribute to impaired proteostasis will provide new avenues for therapeutic interference with the pathogenesis of chronic lung diseases in the young and adult. Here, we introduce the concept of proteostasis and summarize available evidence on dysregulation of proteostasis pathways in pediatric and adult chronic lung diseases.

**Electronic supplementary material:**

The online version of this article (doi:10.1186/s40348-014-0011-1) contains supplementary material, which is available to authorized users.

## Introduction

**Figure 1:**
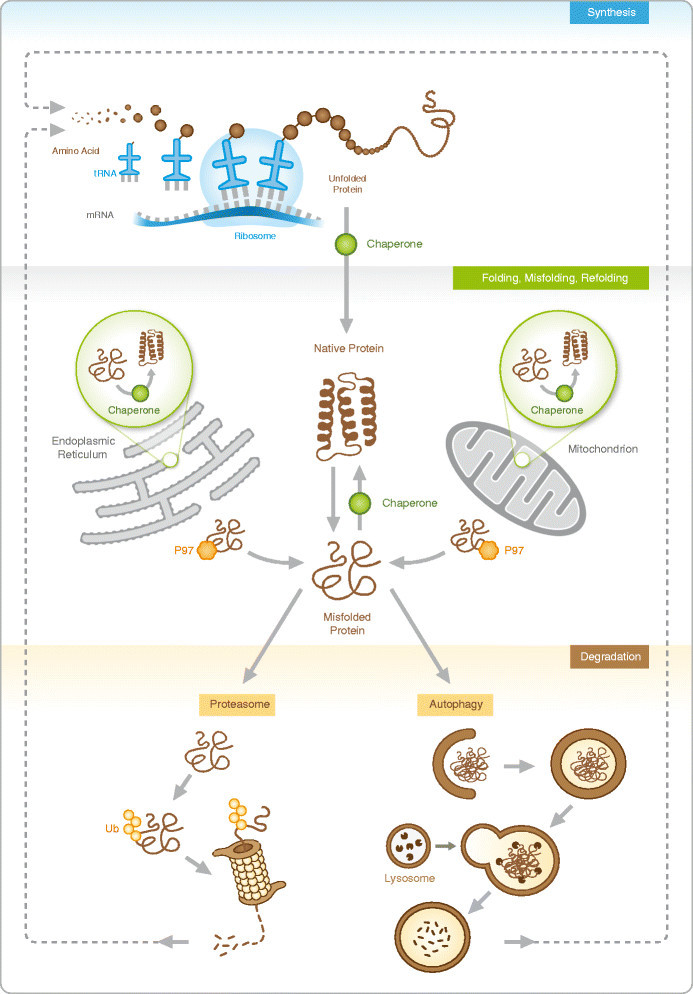
**Life and death of proteins.** Proteins are synthesized as a linear amino acid chain at the ribosomes and fold into their native structure with the assistance of chaperones. Misfolded proteins in the cytosol or in cellular compartments, such as ER and mitochondria, are recognized by chaperones which support their refolding into the native structure. If misfolded proteins cannot be properly refolded, they are targeted for degradation mainly by the ubiquitin-proteasome system. For proteasomal degradation, proteins are tagged with a polyubiquitin chain and subsequently degraded by the proteasome into small peptides. Misfolded proteins of the ER or at the mitochondria are retro-translocated into the cytosol and transported to the proteasome for degradation with the help of VCP/p97. Aggregated proteins are engulfed by the autophagosome and degraded after fusion of lysosomes with the autophagosome. Degradation products of the proteasome and the autophagy pathway are mainly recycled as amino acids for protein synthesis.

Maintenance of a functional proteome and protein interaction network is essential for the survival of cells and organisms [1]. The cellular processes involved in preservation of this protein homeostasis are collectively called proteostasis. It covers all steps in the life of a protein, i.e., protein synthesis, correct protein maturation and folding, as well as the timely disposal of unwanted and damaged proteins by the ubiquitin-proteasome pathway or the lysosome-autophagy route (Figure 1). Dysfunctional proteostasis is emerging as a key and common pathomechanism for chronic lung diseases [2]. The range of lung disorders in which aberrant protein homeostasis has been implicated covers both pediatric and adult lung diseases that occur due to genetic, environmental, or idiopathic causes. The list includes chronic obstructive pulmonary disease (COPD) due to cigarette smoking or alpha-1 antitrypsin (AAT) deficiency, cystic fibrosis, idiopathic pulmonary fibrosis (IPF), pulmonary arterial hypertension (PAH), and some allergic airways diseases. Identifying the pathways of impaired proteostasis is central to interfere with the pathogenesis of chronic lung diseases in the young and adult [3].

## Review

### Protein synthesis, folding, and function

Proteins are synthesized at the ribosome as a linear sequence of amino acids and fold into a complex three-dimensional structure that enables protein function [4]. Protein folding is usually coupled directly to protein translation but also takes place in subcellular compartments, such as the endoplasmic reticulum (ER) or mitochondria, and involves support by molecular chaperones, i.e., heat shock proteins (HSP) [5]. Molecular chaperones also regulate protein localization and protein-protein interactions, thereby contributing to functional protein networks [5]. Stress-induced protein modifications, lack of oligomeric assembly partners, or denaturation results in protein misfolding and challenges protein folding energetics and the cellular chaperone network [6]. Misfolded proteins expose hydrophobic surfaces that may trap other proteins to form protein aggregates, a process called proteotoxicity [6]. Molecular chaperones prevent protein aggregation by binding to these hydrophobic residues, thereby facilitating correct folding in an ATP-dependent manner [5]. Ensuring proper protein folding by molecular chaperone networks thus represents the first level of maintaining a functional proteostasis network. In case chaperones are unable to successfully restore three-dimensional protein structure, misfolded proteins are targeted for proteasomal degradation with the help of specific protein quality ubiquitin ligases [7]. Insoluble protein aggregates are disposed by autophagy (Figure 1).

### Protein degradation by the ubiquitin-proteasome or autophagy pathways

The ubiquitin-proteasome and the lysosomal autophagy pathways are the two main protein disposal systems in the cell. They perform not only housekeeping tasks of normal protein turnover but also protein quality control functions that ensure rapid disposal of misfolded and aggregated proteins.

The autophagy pathway comprises sequestration of cytoplasmic cargo in autophagic vesicles and subsequent degradation in the lysosome. Three different forms of autophagy can be distinguished: chaperone-mediated autophagy in which specific proteins are identified by chaperones and delivered directly to the lysosome, microautophagy which describes internalization of cytoplasmic contents by the lysosome, and macroautophagy for unspecific vesicle-mediated degradation via the lysosome [8]. Macroautophagy, often referred to simply as autophagy, allows disposal not only of bulky protein aggregates but also of dysfunctional organelles or parts thereof. For autophagy, the cargo is sequestered inside a double-membrane vesicle forming an autophagosome which then fuses with lysosomal vesicles to form the autolysosomes where the captured material is degraded by acid hydrolases [8]. Degradation products such as free amino and fatty acids are recycled, thereby constituting the prosurvival function of autophagy, e.g., in response to starvation [8]. On the molecular level, more than 30 autophagy-related genes (ATG) execute all autophagy steps [8]. Impaired nutrient supply as well as multiple forms of cellular stress induce autophagy by regulating expression and activation of ATG [9]. Moreover, autophagy also contributes to innate and adaptive immune surveillance and regulation of host/pathogen responses [10].

Degradation of specific cellular proteins is mainly taken over by the ubiquitin-proteasome system. Specificity of protein degradation is achieved by tagging of degradation-prone proteins with polyubiquitin chains. Polyubiquitination can occur at different lysine residues of the ubiquitin molecules. Differentially linked ubiquitin chains have distinct cellular effects: Lysine (K)48-linked polyubiquitin chains, for example, target proteins for proteasomal degradation while K63 linkages are mainly associated with sorting of proteins for lysosomal degradation pathways [11]. Ubiquitination proceeds along an enzymatic cascade, in which ubiquitin is first activated by the ubiquitin-activating enzyme E1, conjugated by an E2 ubiquitin-conjugating enzyme, and finally covalently linked to the substrate by a specific E3 ubiquitin ligase [11],[12]. Binding of further ubiquitin molecules to the previous one enables formation of a polyubiquitin chain which mediates degradation while modification of proteins with only one ubiquitin molecule mediates signaling [11]. Specific regulation of substrate turnover thus takes place on the level of ubiquitin-conjugating E3 ligases or - as a newly emerging theme - on the level of specific deubiquitinating enzymes, so-called DUBs [13]. The 26S proteasome is the proteolytic unit of the ubiquitin-proteasome system and specifically degrades polyubiquitinated proteins. It is composed of one barrel-shaped 20S catalytic core and one or two cap-like 19S regulatory complexes. While the 19S regulators facilitate binding and deubiquitination, as well as ATP-dependent unfolding of substrates, the 20S proteasome core particle hydrolyzes proteins with the help of three proteolytic active sites into small peptides. The three active sites of the proteasome have different cleavage specificities with β1 preferably cleaving after acid residues, β2 after basic residues, and β5 after hydrophobic residues. The active sites are therefore also named caspase-like, trypsin-like, and chymotrypsin-like active sites, respectively [12]. In immune cells or upon stimulation with interferon-γ, the three standard active subunits are exchanged by inducible subunits to form the immunoproteasome. Degradation products of the proteasome are not only used for recycling of amino acids but also exploited for major histocompatibility complex (MHC) class I antigen presentation to define the ‘cell's self’ towards the immune system [14]. The controlled degradation of cellular proteins is essential for cellular growth, signaling, transcription, immune responses, and protein quality control and places the ubiquitin-proteasome system at the heart of proteostasis [15],[16].

### Organelle-specific proteostasis

Dysfunctional proteins arise in all cellular compartments such as the plasma membrane, cytosol, nucleus, ER, and mitochondria (Figure 1). Misfolded nuclear proteins are degraded by nuclear proteasomes or via nucleophagy [17]. In contrast, as the ER and mitochondria are not accessible for the proteasome, specialized pathways compensate for degradation of misfolded proteins in these compartments. With nearly one third of all proteins being secreted via the ER and requiring complex folding and glycosylation, this organelle is equipped with specialized ER-resident chaperones which recognize misfolded proteins and facilitate their ubiquitination by ER-associated ubiquitin ligases and subsequent transport into the cytosol for ubiquitin-mediated proteasomal degradation. This process is dependent on the AAA ATPase VCP/p97 and is termed ER-associated degradation pathway (ERAD) [18]. A similar pathway exists for mitochondrial proteins, i.e., mitochondria-associated degradation (MAD) which involves disposal of mitochondrial proteins - mainly from the outer membrane - via a VCP/p97 and proteasome-dependent pathway [19].

In addition, misfolded proteins in these compartments trigger a compartment-specific unfolded protein response (UPR) which aims to adjust the capacity of folding and disposal in the organelle [20],[21]. Sequestering of chaperones by misfolded proteins in the ER is rapidly sensed by three sensor proteins: IRE1, PERK, and ATF6, and launches the three-armed adaptive stress response of the ER. While activation of PERK attenuates protein translation thereby decreasing protein reloading of the ER, IRE1 and ATF6 concertedly activate UPR-specific transcription to increase the ER folding capacity and function. When they fail, however, e.g., upon severe or chronic ER stress, apoptosis is induced [22]. Accumulation of misfolded proteins in the mitochondrial matrix impairs the import of the transcription factor ATFS-1 into mitochondria but triggers its translocation to the nucleus to induce expression of mitochondrial proteases and chaperones [21]. A distinct mitochondrial unfolded protein response exists also for the intermembrane space [23]. Organelle proteostasis also involves bulk degradation of whole organelles via the autophagy pathway such as nucleophagy or mitophagy or by autophagic disposal of parts of the dysfunctional ER [17],[24].

### Proteostasis in chronic lung diseases

Dysfunctional proteostasis is a common pathomechanism for both, pediatric and adult, chronic lung diseases that either are caused by inherited protein dysfunction or involve sporadic impairment of protein homeostasis [2]. In this section, we will summarize available knowledge on proteostasis in the young and immature as well as in the adult and aged lung.

#### Hereditary dysfunction of proteostasis

In the lung, the two major inherited genetic disorders, cystic fibrosis and AAT deficiency, are conformational disorders that are caused by the hereditary expression of mutant alleles of the cystic fibrosis transmembrane conductance regulator (CFTR) and AAT, respectively [25]. In addition, mutations of the surfactant proteins (SP) C, B, and A have been identified in familial cases of IPF [3],[26]. All of these mutations pose a serious challenge for the proteostasis network as they result not only in impaired function of the respective protein but also in ER stress, dysregulated proteasome, and altered autophagy function [25]-[27]. The deltaF508CFTR mutant - the most predominant CFTR mutation in cystic fibrosis patients - is characterized by a misfolded CFTR protein which is rapidly degraded by the proteasome and thus fails to translocate to the plasma membrane [28]. Consequently, modulating the proteostasis network to stabilize folding and trafficking of this CFTR mutant was suggested as a beneficial therapeutic approach in CF [29]. Loss of functional CFTR has further been associated with defective autophagy and accumulation of aggregated and polyubiquitinated proteins. Restoration of autophagy increased CFTR trafficking to the cell membrane [15],[30]. There is also evidence for enhanced ER stress in CF patients as summarized elsewhere [25], and inactivation of the XBP-1 pathway decreased inflammatory cytokine production in a model of inflamed CF airway linking ER stress to inflammation [31]. Likewise, in AAT deficiency, misfolded AAT variants fail to exit the ER and are targeted for ERAD. Some AAT variants polymerize and are degraded via autophagy [25]. Thereby, misfolded AAT accumulates in hepatocytes and cannot be secreted in the bloodstream, which leads to unopposed protease activity in the lung [30]. Activation of autophagy prevents aggregate toxicity in hepatocytes and reversed liver pathology in a mouse model of AAT deficiency [30]. Misfolded AAT primarily leads to proteostasis imbalance and ER stress mainly in the liver while the lung phenotype manifests later in life due to unopposed protease activation [25]. However, as aggregates of mutant AAT were also detected, e.g., in alveolar macrophages, enhanced aggregate clearance by autophagy might also be beneficial in the lung [30]. Similarly, most of the known mutations of the SP-C and SP-A genes result in abnormal processing, folding, and accumulation of the mutant protein in the ER, possibly triggering ER stress in alveolar epithelial type II cells (AECII). Subsequent induction of apoptosis in the alveolar epithelium may then initiate fibrotic tissue remodeling and chronic inflammatory responses [26],[32]. In addition to ER stress, dysfunction of the proteasome and autophagy pathways has been shown for some of the SP-C mutants *in vitro*[33]. The rare hereditary disorder Hermansky-Pudlak syndrome is also characterized by disturbed proteostasis, namely impaired biogenesis of lysosome-related organelles, which contributes to continuous damage and apoptosis of AECII and early onset of pulmonary fibrosis [34].

#### Sporadic dysfunction of proteostasis

In addition to hereditary conformational disorders of the lung, proteostasis is challenged by exposure of the lung to noxious particles, chemicals, allergens, and pathogens which starts early in life and continues throughout life, thereby contributing to age-related chronic lung diseases [27],[35].

##### Impaired proteostasis in the young and immature lung

Mechanical ventilation and oxygen supply are often used as a treatment in preterm-born infants. While lifesaving, both, mechanical hyperinflation and hyperoxia, damage the immature lung, thus contributing to development of chronic lung diseases such as bronchopulmonary dysplasia (BPD) which persists during childhood and adulthood [36]. Among others, mechanical ventilation and hyperoxia induce formation of reactive oxygen species (ROS) which challenge the proteostasis network of the immature lung due to oxidative protein modifications and protein folding stress [37],[38]. Thus, exposure of newborn mice to hyperoxia induced expression of ER stress markers including CHOP that may contribute to increased apoptosis [39]. Furthermore, hyperoxia altered ER morphology and eIF2α phosphorylation but did not affect other UPR pathways in a newborn rat model [40]. Whether mechanical ventilation and hyperoxia also trigger autophagy or proteasome dysfunction in infants is largely unknown. In adult mice lungs, however, autophagy is increased upon mechanical ventilation and contributes to cellular damage [41]. Furthermore, hyperoxia induced autophagosome formation and expression of autophagic proteins in bronchial epithelial cells and in mice. Enhanced expression of the autophagic protein LC3B-II decreased apoptosis in hyperoxia-treated cells by regulating the extrinsic apoptosis pathway [42]. In hyperoxia-treated adult mice, elevated levels of PINK1 helped to maintain mitochondrial homeostasis by inducing autophagy of damaged mitochondria. In this study, higher activity of the lung proteasome was observed as well [43]. Hyperoxia also augmented ubiquitination and expression of the chaperone HSP70 in bronchial epithelial cells from adult patients treated with >95% oxygen [44]. In contrast, HSP27 expression was decreased in hyperoxia-treated newborn rats and in a human alveolar epithelial cell line, A549 cells, while overexpression of HSP27 in A549 cells attenuated hyperoxia-induced cell death [45]. In summary, there is rising evidence for dysfunctional proteostasis in the ER and cytosol in response to mechanical ventilation and/or hyperoxia in the adult and also immature lung.

Asthma is the most common respiratory lung disease in children affecting more than 10% of children between 2 and 15 years of age [46]. Recent studies support the concept of asthma, although primarily an inflammatory disease, as a disease initiated by recurrent dysfunction of the airway epithelium which modulates inflammatory responses [47]. Accumulating evidence suggests a key role for oxidative events and protein damage in the pathogenesis of bronchial asthma [48],[49]. Accordingly, ER stress markers were found to be increased in blood cells and bronchoalveolar lavage (BAL) from asthmatic patients and in the lung tissue of ovalbumin-challenged mice. Furthermore, administration of chemical chaperones reduced experimental asthma and inflammation in mice [50]. This effect was mainly mediated by attenuating ER stress-induced NFκB activation [50]. As NFκB activation can also be attenuated by inhibition of the proteasome, proteasome inhibitors have also been suggested as potential therapeutics in asthma. However, although proteasome inhibitors had some beneficial anti-inflammatory effects, they were unable to ameliorate chronic asthma in mice [16]. In addition, autophagy was shown to be augmented in asthmatic patients: Expression of Atg5 was increased in nasal mucosal cells of children with acute asthma and was associated with a single nucleotide polymorphism (SNP) in the Atg5 promoter [51]. A second SNP in the ATG5 gene was also associated with the enhanced formation of autophagosomes [52]. Thus, some initial evidence implicates an involvement of autophagy in asthma, but the molecular mechanisms remain to be elucidated.

Another aspect of how impaired proteostasis may affect development of asthma relates to the role of the proteasome in MHC class I antigen presentation: As the peptide products of proteasomal protein degradation may be used for MHC class I antigen presentation, proteasome function is an essential part of the host response towards intracellular infections by activating CD8+ T cell-mediated adaptive immunity against viral infections [14]. Only one study has addressed the putative involvement of the immunoproteasomes in asthma development using knockout mice for the immunoproteasome subunit LMP7. LMP7-deficient animals mounted a strong Th2 response during ovalbumin-induced but not house dust mite-induced acute asthma [53]. The role of immunoproteasome-dependent immune responses to virus infection in asthma has not been investigated so far. In general, the contribution of proteostasis systems to sporadic lung disease in infants and children is only beginning to be unraveled. Recent evidence shows that protein homeostasis is dysregulated in the development of BPD and asthma. However, to what extent the different proteostasis pathways contribute to these pediatric diseases, how they interact or compensate for each other, and whether they represent a feasible therapeutic target await further investigation.

##### Deficient proteostasis in the adult and aged lung

Impaired proteostasis has been identified as a key and common pathomechanism for sporadic chronic lung diseases as reviewed extensively elsewhere [2],[15],[16],[30]. Thus, we will only briefly outline the basic findings and would like to refer the reader to the details as given in the above cited reviews.

For sporadic chronic lung diseases in the adult, cigarette smoke remains the main risk factor. As such, cigarette smoke exposure of the lung represents a paradigm for environmental insults that drive pathogenesis of chronic lung diseases. Among the more than 4,700 chemicals of cigarette smoke, reactive compounds oxidatively modify cellular proteins, thereby seriously challenging the proteostasis network of the cell. Several proteins such as secretory proteins, histone-modifying enzymes, signaling mediators, and transcriptional regulators have been shown to be oxidatively modified by cigarette smoke, adding to cellular dysfunction and pathogenesis of chronic lung diseases [54],[55]. In agreement with the increased burden of misfolded proteins, e.g., upon cigarette smoke exposure, protein quality control pathways are mainly activated in adult chronic lung diseases. Induction of autophagy is a prominent feature in the lungs of patients with COPD as well as in mice chronically exposed to cigarette smoke [56]. Autophagy is also increased in PAH tissue, and mice lacking the autophagic protein LC3B showed exaggerated features of PAH in a hyperoxia model [30],[57]. These findings together with the observation of fragmented mitochondrial morphology in pulmonary smooth muscle cells of PAH patients raised some speculation on a pathogenetic contribution of mitophagy in PAH [58]. However, activation of mitophagy in PAH could not be confirmed experimentally. Conflicting data also exist for the role of mitochondrial quality control in COPD. In IPF, autophagy pathways are not activated but rather impaired [59]. A recent study as well as unpublished data from our lab suggests that proteostasis in IPF might be rather regulated on the level of 26S proteasome activation [3],[60]. For COPD, conflicting data exist on proteasome function in the lung: While Malhotra et al. observed a decline in proteasome expression and activity in the lungs of COPD patients which correlated with progressive disease severity [61], Baker et al. did not observe any decline in proteasome activity in end-stage COPD tissue [60]. Induction of ER stress in the lung has been observed in response to cigarette smoke exposure as well as in pulmonary fibrosis [62]. In transgenic mouse models, induction of ER stress in the alveolar epithelium predisposes to enhanced lung fibrosis after treatment with bleomycin [26]. Furthermore, ER stress markers are observed in pulmonary hypertension, and treatment of rats with the modulator of the unfolded protein response salubrinal attenuated experimental PAH [63].

Protein quality control pathways are not only activated as an adaptive response to altered proteostasis, but they may also be direct targets of noxious environmental exposures as recently shown for the proteasome. Acute exposure to cigarette smoke directly inhibits the catalytic activity of purified proteasomes and proteasome activity of bronchial and alveolar epithelial cells as well as in mouse lungs [64],[65]. Oxidative impairment of the ER-resident protein disulfide isomerase by cigarette smoke also expands protein misfolding in the ER compartment [54]. Such impairment of protein quality control further exacerbates the detrimental effects of noxious exposures of the lung and may amplify progression of chronic lung diseases. In line with this notion, proteostasis has been shown to decline with aging [66]. As a consequence of aging, misfolded proteins accumulate over time and proteostasis pathways become functionally impaired in response to persistent or repeated environmental exposures, thereby amplifying protein damage [6]. Age-related impairment of proteostasis has been observed on all levels including diminished chaperone availability, altered autophagy regulation, impaired proteasome function, and activation of unfolded protein responses in the ER and mitochondria [66]-[68]. Of note, only very few studies analyzed the impact of an aged proteostasis network on the development or progression of chronic respiratory diseases using animal models [69]. Further research will be required to delineate the contribution of single proteostasis pathways to distinct age-related chronic lung diseases.

## Conclusions

Dysregulation of proteostasis is an emerging common pathomechanism for chronic lung diseases in the adult and aged patient. There is also rising evidence that sporadic impairment of protein homeostasis contributes to early disease onset in pediatric lung disorders beyond the well-known hereditary proteostasis disorders such as cystic fibrosis and AAT deficiency. Maintaining protein homeostasis in the lung is particularly relevant in any condition that leads to sustained generation of ROS, e.g., mechanical ventilation, hyperoxia, and exposure to noxious particles and gases. In particular, compartment-specific dysregulation of proteostasis such as the unfolded protein response of the ER and mitochondria and altered proteasome function are emerging as novel aspects of dysregulated proteostasis in the diseased lung. The interplay of the proteostasis network with other essential cellular functions such as cilia and vesicle transport is only beginning to be unraveled [3]. In particular, the close interaction of protein homeostasis with immune response pathways is a largely neglected aspect of how impaired proteostasis within a single cell outreaches its neighborhood: Proteasome function is essential for MHC class I-mediated adaptive immunity against viral and intracellular infections [14]. In addition, autophagy is central for innate immune surveillance of pathogens and also affects MHC class II presentation [70]. Moreover, proper function of the ER is required to ensure expression of immune relevant surface molecules and secretion of cytokines [71].

This adds a novel aspect to the concept of modulating proteostasis for disease intervention as proposed by Bouchecareilh and Balch [27] and may stimulate further research on dysregulated proteostasis in the young and adult lung.

## Authors’ original submitted files for images

Below are the links to the authors’ original submitted files for images. Authors’ original file for figure 1

## Abbreviations

AAT: alpha-1 antitrypsin
AECII: alveolar epithelial type II cells
ATG: autophagy-related genes
BAL: bronchoalveolar lavage
BPD: bronchopulmonary dysplasia
CFTR: cystic fibrosis transmembrane conductance regulator
COPD: chronic obstructive pulmonary disease
ER: endoplasmic reticulum
ERAD: ER-associated degradation
IPF: idiopathic pulmonary fibrosis
MAD: mitochondrial-associated degradation
MHC: major histocompatibility complex
PAH: pulmonary arterial hypertension
ROS: reactive oxygen species
SP: surfactant protein
UPR: unfolded protein response
