# Supplementary figures and images for: Proteostasis in pediatric pulmonary pathology
Source: Mol Cell Pediatr. 2014 Dec 29;1:11. doi: 10.1186/s40348-014-0011-1 (PMC4530569; doi:10.1186/s40348-014-0011-1)

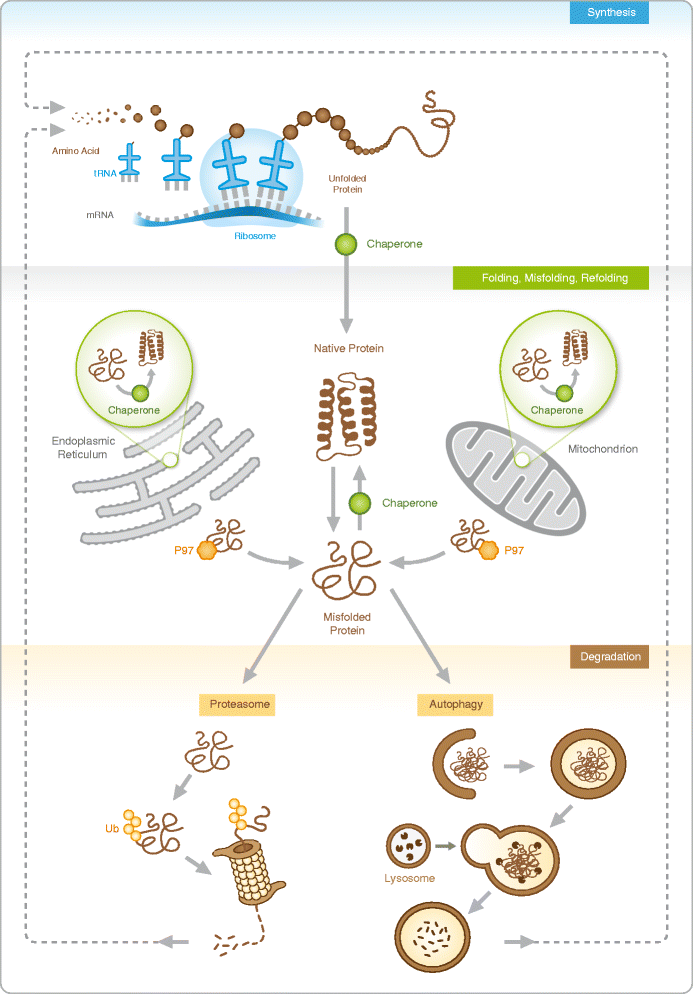

Supplement: Supplementary file 1 — Authors’ original file for figure 1 [file 40348_2014_11_MOESM1_ESM.gif]
